# Design of Thermosensitive Niosomes by Eutectic Mixture of Natural Fatty Acids

**DOI:** 10.3390/pharmaceutics16070909

**Published:** 2024-07-07

**Authors:** Elisabetta Mazzotta, Martina Romeo, Zakaria Hafidi, Lourdes Perez, Ida Daniela Perrotta, Rita Muzzalupo

**Affiliations:** 1 Department of Pharmacy, Health and Nutritional Sciences, University of Calabria, Via P. Bucci, 87036 Arcavacata di Rende, Italy; martina.romeo@unical.it; 2 Department of Surfactants and Nanobiotechnology, Institute for Advanced Chemistry of Catalonia (IQAC-CSIC), 08034 Barcelona, Spain; zhatnt@cid.csic.es (Z.H.); lpmste@cid.csic.es (L.P.); 3 Centre for Microscopy and Microanalysis (CM2), Department of Biology Ecology and Earth Sciences, University of Calabria, 87036 Arcavacata di Rende, Italy; ida.perrotta@unical.it

**Keywords:** phase change materials, natural fatty acids, niosomes, thermoresponsive release, antibacterial activity

## Abstract

In the current study, a smart release system responsive to temperature was developed to improve the efficiency of tetracycline (TC) in antibacterial therapy. The nanovesicles designed consist of a non-ionic surfactant, SPAN60, cholesterol and a phase change material (PCM) as a thermoresponsive gating material. Niosomes were prepared using an increasing amount of PCM and characterized in terms of size, zeta potential, colloidal stability and thermoresponsive properties. The vesicles that developed were homogenous in size, had good biocompatibility and stability for up to 3 months and demonstrated thermoresponsive behavior. A low drug leakage was observed at 37 °C, while a rapid release occurred at 42 °C, due to the faster diffusion rate of the drug trough the melted PCM. This controllable drug release capacity allows us to avoid premature drug release, minimizing unwanted and toxic effects and ensuring a long retention time in the nanodevice so that it reaches the infected sites. In addition, TC-loaded niosomes were screened to investigate their antibacterial activity against various Gram-positive and Gram-negative bacteria by minimum inhibitory concentration (MIC) and minimum bactericidal concentration (MBC) assays. An interesting temperature-dependent antibacterial activity was observed against some bacterial strains: the niosomes activity against *S. epidermis*, for example, was improved by the temperature increase, as suggested by a reduction in MIC values from 112.81 to 14.10 μM observed at 37 and 42 °C, respectively. Taken together, the thermoresponsive platform developed allows us to use lower antibiotic amounts while ensuring therapeutic efficacy and, so, will advance the development of a novel antibacterial agent in clinical practice.

## 1. Introduction

Currently, infectious diseases are a serious and critical threat to global health, representing the major cause of morbidity and mortality. Several factors contribute to the ineffectiveness of existing antibiotics, such as low water solubility, low bioavailability and stability, low patient compliance as a result of frequent administration and the rise of adverse side effects [1]. Furthermore, the excessive and improper use of conventional antibiotics has led to the emergence and rapid spread of multidrug-resistant (MDR) bacteria, such as *Escherichia coli*, *Klebsiella pneumoniae* and *Staphylococcus aureus*, that do not respond to standard treatments [2].

The development of new effective and safe antibacterial agents is a complex and expensive process that occur over the long term. Consequently, new strategies have been developed to enhance the effectiveness of existing antibiotics. Recently, nanotechnology has represented an attractive method of improving the therapeutic effects of common antibiotics [3]. Nano-sized structures can act as antibacterial agents by themselves or as carriers of existent antimicrobials to promote bioavailability and effectiveness [4]. Nanocarriers are able to improve the solubility of hydrophobic antibiotics, enhance cell membranes’ permeability and sustain antibiotic release and intracellular concentrations [5,6]. Notably, a few formulations based on liposomes (AmBisome^®^, Abelcet^®^ and Amphotec^®^) have been already approved by regulatory agencies for use in human patients and have exhibited better pharmacokinetics than conventional formulations, including prolonged systemic-circulation half-life, enhanced therapeutic efficacy and, most importantly, reduced side effects [7]. Various factors, such as chemistry, particle size and shape, surface-to-volume ratio and zeta potential, affect antimicrobial activity [2]. However, the short shelf-life and stability, encapsulation efficacy and rapid clearance from the blood stream limit the efficacy of liposomes for antimicrobial treatment.

Recently, stimuli-responsive antimicrobial nanoparticles have gained considerable importance, as they have overcome some important challenges in the treatment of infections. This is because they recognize and respond dynamically to specific internal stimuli associated with the pathological infection microenvironment or to external physical stimuli obtained by applying an external source [8]. Targeted drug release allows us to increase the local concentration of the active substance at the infection site, minimizing the accumulation in healthy tissue and reducing the risk of systemic side effects [9]. Both chemical and physical stimuli can be used to induce conformational changes in these smart antibiotic devices that deliver antibiotics to the infection site in a controlled way [10]. Nanocarriers, indeed, can be tailor-made, designed to possess a wide range of chemical functionalities that easily undergo hydrolysis, enzymatic degradation or conformational changes in response to pH, redox status, temperature, light, ultrasound and proteolytic enzymes. Among them, temperature is the most widely used external stimulus to induce drug release in smart nanocarriers. Typically, these formulations contain in their structure molecules that change their physical properties in response to temperature alterations [11]. Therefore, drug release can be tailored to be slow and steady under physiological conditions or fast when the temperature increase.

Various molecules can be used to achieve temperature-controlled release, such as polymers, surfactants, phospholipids and phase change materials [12,13]. Specifically, phase change materials (PCMs) are materials with a high latent heat of fusion that undergo the solid–liquid or liquid–solid transition in response to narrow temperature changes [14]. PCMs are promising tools used to construct smart delivery systems for their release and control, as, at transition temperatures, they melt and release drugs quickly [15]. Consequently, this responsivity to specific temperatures allows us to control the release of drugs at the target site, avoiding premature drug leakage, improving therapeutic efficacy and reducing harmful side effects. Among the various types of PCMs available, those of particular interest include natural fatty acids for their low toxicity, easy biodegradation and low cost [16,17]. Due to the limited types of natural fatty acids, it is difficult to create PCMs with melting points close to the physiological temperature using a single component [14]. Therefore, researchers proposed to employ two or more kinds of fatty acids in several ratios to overcome these drawbacks and to achieve controllable melting temperatures [18]. In this light, this study has been conceived to design smart thermoresponsive niosomes based on eutectic mixtures of natural fatty acids for antibiotic delivery. For this purpose, a eutectic mixture of natural fatty acids composed of lauric acid and stearic acid was used as a thermoresponsive gate in the vesicle bilayer. In fact, the eutectic mixture, at the weight ratio of lauric acid (mp = 44 °C) and stearic acid (mp = 69 °C) 4:1, exhibits a sharp melting point at 39 °C, and can be used for thermoresponsive targeting [15]. This PCM was incorporated in the niosome bilayer based on Span 60 and cholesterol (Ch) in order to control the release of a broad-spectrum antibiotic, Tetracycline hydrochloride (TC), which is active against both Gram-positive and Gram-negative bacteria. Commonly, TC efficacy is seriously limited by its low bioavailability, side effects and the emergence of resistance [19,20]. Consequently, its encapsulation in a smart nanodevice, as proposed in this work, could be a potential solution to improve its clinical efficacy.

## 2. Materials and Methods

Lauric acid, stearic acid, cholesterol (Ch), sorbitan monostearate (Span^®^ 60) and tetracycline hydrochloride (TC) were purchased from Sigma-Aldrich (Milan, Italy). Mueller-Hinton Broth (MH) was purchased from Difco Laboratories (Detroit, MI, USA). All solvents were of analytical grade and used without further purification.

### 2.1. Preparation of Thermoresponsive Niosomes

First, lauric acid and stearic acid (4:1 by weight) were dissolved in chloroform to prepare the PCM. Various niosome formulations were developed by modulating the molar ratio of Span60, Ch and PCM using the thin-film hydration method. For this purpose, Span60 and Ch were solubilized in ethanol-and-chloroform mixed solvents (*v/v* 4:1). The PCMs were added directly to the solubilized surfactant, and the final solution was then dried in a rotary evaporator at 40 °C to obtain a thin lipid film. The lipid film was then hydrated with 10 mL TC aqueous solution (8.31 × 10^−4^ M, pH 6.8) for 20 min at 60 °C. After that, the dispersions obtained were firstly cooled at room temperature for 5 min and then at 4 °C in an ice water bath for 10 min, accompanied by mechanical stirring to rapidly solidify the fatty acid eutectic mixture and promote the encapsulation of the drug.

### 2.2. Characterization of Thermoresponsive Niosomes

The size and zeta potential of thermoresponsive niosomes were evaluated by dynamic light scattering (Malvern, Zetasizer Nano ZS) at a scatting angle of 90°. The morphology was characterized by transmission electron microscopy (HITACHI, Ht-7700, Japan).

The encapsulation efficiency (EE%) of TC in the nanovesicles was evaluated after purification by a dialysis method for 4 h at 25 °C. The purified niosomes were diluted to 1:100 (*v/v*) using ethanol in order to break the niosomal bilayers and to release the encapsulated compounds. The concentration of TC was measured using a UV−vis spectrometer at 271 nm and calculated according to a standard curve. The EE was calculated using Equation (1):

(1) EE %=(TC in purified niosomes)/(TC c in  non purified niosomes) ×100

The stability of thermosensitive vesicles was evaluated by determining the sizes and zeta potentials at specific time points at up to 3 months. Each measure was repeated three times, and the results were recorded as the mean ± standard deviation.

Moreover, we sought to examine the in vitro stability of niosomes in PBS, NaCl 0.9% and MH medium, since these conditions would affect the particle size and consequently the stability, uptake and antibacterial efficacy. To perform this preliminary biological evaluation, 0.5 mL of the niosomes was diluted in up to 5 mL, and the average size, polydispersity index and ζ-potential were evaluated by DLS, maintaining samples at 37 °C and performing measurements at different time points (0, 0.5, 2, 5, 24, 48 and 120 h).

Thermograms of the physical mixture of Span60, CH and PCM mixtures were recorded using differential scanning calorimetry (DSC SETARAM 131). The samples were placed in an aluminum pan and scanned at a rate of 3 °C/min over a temperature range of 20–85 °C under a purge of nitrogen.

### 2.3. In Vitro Thermoresponsive Drug Release

To evaluate the thermoresponsivity of developed niosomes, antibiotic release experiments were investigated by a dialysis method at two different temperatures (37 and 42 °C). Typically, 1 mL of TC niosomes solution was poured into a dialysis bag (MWCO = 12–14 kDa), which was immersed in 25 mL of distilled water in a thermostat bath. At certain time intervals, 2 mL of solution was taken out and replaced with 2 mL of a fresh buffer. The absorbance value of the release solution was recorded at 271 nm using a UV–vis spectrophotometer, and the drug concentration was calculated according to the standard curve of TC. The results are reported as the cumulative release (%) using the following equation:

TC Release %=(TC release from niosomes)/(initial TC in niosomes) ×100

### 2.4. Analysis of Drug Release Kinetics

The mechanism of TC release for each formulation was exploited by fitting release data to different kinetic models, including first-order, Korsmeyer–Peppas, Peppas–Sahlin and Weibull models [21], using the mathematical equation reported in Table 1:

Where Q represents the amount of drug released at time t; Q_0_ is the initial amount of the drug; and k is the release constant. The applicability of the release kinetics models was evaluated considering the regression coefficient of correlation (r^2^), Adj. R-Square, Pearson’s r and residual sum of squares. A higher value of Adj. R-Square and Pearson’s r indicate a model applicable to the release curve [22]. Conversely, a lower residual sum of squares value highlights the better kinetic model [23].

### 2.5. Hemolysis Assay

Erythrocyte Rabbit blood samples were provided by the animal facility of the Research and Development Center (CID)–Spanish National Research Council (CID-CSIC, Barcelona, Spain). The collection of samples strictly followed the bioethical guidelines outlined by Spanish legislation, which follows the international agreements on that matter under a project authorized by competent authorities (license number: 9821). The project was conducted in a licensed center (the Animal Facility of CID) by accredited researchers under the conditions established in article 15.3 of RD 53/2013. It has been designed according to the 3R principle. Appropriate measures have been taken to avoid unnecessary pain and minimize animal suffering.

Following the method described by Pape et al. [24], red blood cells were washed three times with a solution of PBS pH 7.4. Different volumes of a niosomal solution (1190 μg/mL/5940.5 μM), ranging from 10 to 100 μL, were placed in an Eppendorf, to which 25 μL of erythrocyte suspension and phosphate-buffered saline were added for a total volume of 1 mL. The samples were mixed for 10 min at room temperature and then centrifuged for 5 min at 10,000 rpm. The percentage of hemolysis was determined by comparing the absorbance at 575 nm of the supernatant of the samples with that of the control, which was totally hemolyzed with distilled water. Each analysis was performed in triplicate.

### 2.6. In Vitro Antibacterial Activity

To evaluate the antimicrobial activity of the thermosensitive niosomes, the MIC, defined as the lowest concentration of antimicrobial agents that inhibits the development of visible growth after 24 h of incubation at 37 °C, was determined in vitro. The next representative Gram-positive and Gram-negative bacteria were tested: *Bacillus subtilis* ATCC 6633, *Staphylococcus epidermidis* ATCC 12228, *Staphylococcus aureus* ATCC 29213, *Listeria monocytogenes* ATCC 15313, *Enterococcus faecalis* ATCC 29212, *Escherichia coli* ATCC 25922, *Acinetobacter baumannii* ATCC 19606 and *Klebsiella pneumoniae* ATCC 13048. These strains were from the DSMZ-German Collection of Microorganisms and Cultures GmbH (Inhoffenstraße 7B, 38124 Braunschweig, Science Campus Braunschweig-Süd GERMANY). The MIC values of the developed niosomes were determined using a broth microdilution method; these types of methods are now considered the international reference for susceptibility testing methods [25]. The test was carried out in microdilution plates with a capacity of 200 µL and followed the EUCAST DISCUSSION DOCUMENT E. Dis 5.1 [26]. The broth microdilution assay was performed at two different temperatures. The inoculum was prepared by diluting an overnight broth culture from an agar medium in MH broth and adjusted to give a turbidity equivalent to the McFarland 0.5 standard (1.5 × 10^8^ cfu/mL). Serial dilutions of niosomes in Mueller–Hinton broth (MHB) were prepared, and 200 μL of this was dispensed into the corresponding wells of a 96-well polypropylene microtiter plate. Then, an initial culture of each bacterial strain was added to obtain a final inoculum of about 5 × 10^−5^ colony forming units (CFU)/mL. The positive control wells contained MHB and the bacterial suspension without the niosomes, whereas the negative control wells contained MHB and the niosomes without bacterial suspension. Multi-well plates were incubated after sample inoculation at two different temperatures, 37 °C and 42 °C. After 24 h, the MIC was visually determined. The development of turbidity in an inoculated medium is a function of growth, and reflects an increase in both mass and cell number. It was also checked that there was a sufficient growth of bacteria in the positive control and no growth in the uninoculated control. The turbidity in each well was compared with that of the positive control, and the MIC was recorded as the lowest concentration of the compound that completely inhibits the growth. To confirm the MIC observation, 20 μL of 0.015% *w*/*v* resazurin was added to each well and was allowed to react for about 2 h with the bacteria at the two temperatures. Resazurin was reduced to resorufin by the aerobic respiration of metabolically active cells. As a result, a color change from blue to pink is representative of the presence of viable cells, and with them, this compound can be used to detect the presence of live bacteria. After the incubation period, the bacterial growth indicator changed from blue to pink, confirming the MIC value. Then, the MBC (the antimicrobial concentration corresponding to at least a 3-log reduction in viable cells) was determined. A 10 μL aliquot of the MIC well and the two immediately preceding concentrations was seeded on Muller–Hilton agar and incubated for 24 h at 37 °C. The MBC was determined as the lowest concentration at which no colonies were observed on the agar plates.

## 3. Results and Discussion

### 3.1. Physicochemical Characterization

The aim of this work was to develop a thermosensitive nanodevice for localized antibiotic delivery. Unspecific antibiotic delivery has, indeed, important side effects, such as sub-lethal concentrations at the site of infection, a lack of targeting, causing the disruption of intestinal flora, and the appearance of multidrug resistance. The encapsulation of antibacterial agents in niosomes has been widely pursued in recent years to improve their stability and biocompatibility, prolong their release and enhance their antibacterial efficacy [27].

Niosomes are formed through the self-assembly of non-ionic surfactants in water, creating closed bilayers that can encapsulate both lipophilic and hydrophilic substances within the bilayer membrane or the aqueous layer, respectively. This assembly process depends on the hydrophilic–lipophilic balance (HLB), critical packing parameter values, and gel–liquid transition temperature of nonionic surfactant, and typically requires energy input, such as physical agitation or heat. Compared to liposomes, niosomes are more stable, and the presence of non-ionic surfactants allows for their prolonged circulation, potentially enhancing therapeutic efficacy and targeted delivery [28]. Additionally, the phospholipids used in liposome manufacturing are both expensive and prone to degradation. These factors make niosomes a more stable and cost-effective option for drug delivery systems [29].

Considering the recent success of stimuli-responsive nanocarriers to improve antibiotic efficacy, we focused on the design of thermoresponsive niosomes for the control of TC release. For this goal, a eutectic mixture of lauric and stearic acid is used as a thermoresponsive gatekeeper of the vesicle bilayer. The choice of this type of fatty acid was mainly due to the fact that, when combined at a weight ratio of 4:1, it has a well-defined melting point at 39 °C.

Niosomes were prepared via a film hydration approach by changing the molar ratios of non-ionic surfactant, CHOL and PCM (Table 2) in accordance with the goal of obtaining a stable formulation at physiological temperatures and a higher release in hyperthermic conditions.

**Figure 1:**
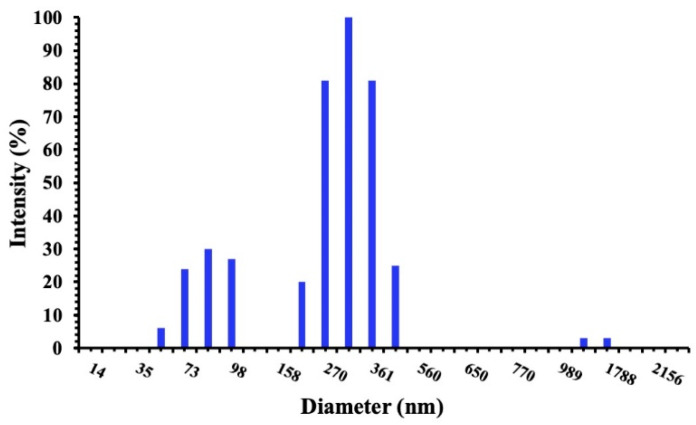
Intensity size distribution of SP60CHPCM2 by DLS analysis.

When visually observed, the niosomes appeared as milky and turbid dispersions, which are the typical aspect of vesicular formulations. The physicochemical properties of vesicles are reported in Table 2. The mean particle sizes ranged between 244.9 and 348.3 nm, with a P.I. lower of 0.3 indicating a homogeneous and narrow size distribution (Figure 1).

The increase in cholesterol content resulted in a decrease in vesicle size, since surface free energy usually decreases with increasing hydrophobicity, and therefore resulted in smaller vesicles [30]. A similar trend was also observed with the increase in PCM content in vesicle bilayers, which further raises nanocarrier hydrophobicity. The formulations developed presented a good ability to encapsulate TC in an aqueous core, as suggested by the high values of EE% that ranged between 53.44 and 81.29 (Table 2).

The drug loading led to an important variation in particle size: a significant increase in the hydrodynamic diameter was indeed observed (Table 3). This trend is already reported in the literature and is due to the fact that TC has a negative charge and, when it was located in the aqueous compartment, was free to move and cause an electrical repulsion to each other, leading to an increase in vesicle size [31,32].

Despite its non-ionic character, the SP60 niosomes showed a large negative Z-potential value. The incorporation of the fatty acid mixture gave rise to lower Z-potential values due to the negative charge of these compounds. Additionally, all formulations exhibited large negative Z-potential values, which help to increase the stability of these formulations due to electrostatic interactions. The TC-loaded niosomes (Table 3) had higher values, but they were still negative enough to maintain their stability. All these niosomes (free and TC-loaded) presented low polidispersion indexes (0.175–0.281), which can also contribute to enhancing the stability of these aggregates.

**Figure 2:**
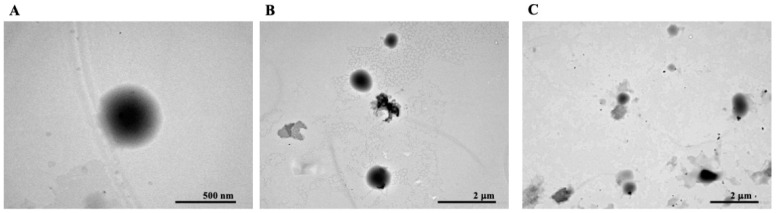
Representative TEM images of (**A**) a single vesicle and (**B**,**C**), a population of SP60CHPCM2 vesicles. Scale bars are (**A**) 500 nm, (**B**) 2 μm and (**C**) 2 μm.

The size and morphology of the SP60CHPCM2 niosomes have also been studied using TEM. These niosomes showed the best in vitro cumulative release profile of TC (see the next section) because it was chosen to carry out these studies. As shown in Figure 2, the morphology of SP60CHPCM2 niosomes results is spherical (Figure 2A), and the shape was homogeneous throughout the whole population (Figure 2B,C). Moreover, the sizes were concordant with that evaluated by DLS.

**Figure 3:**
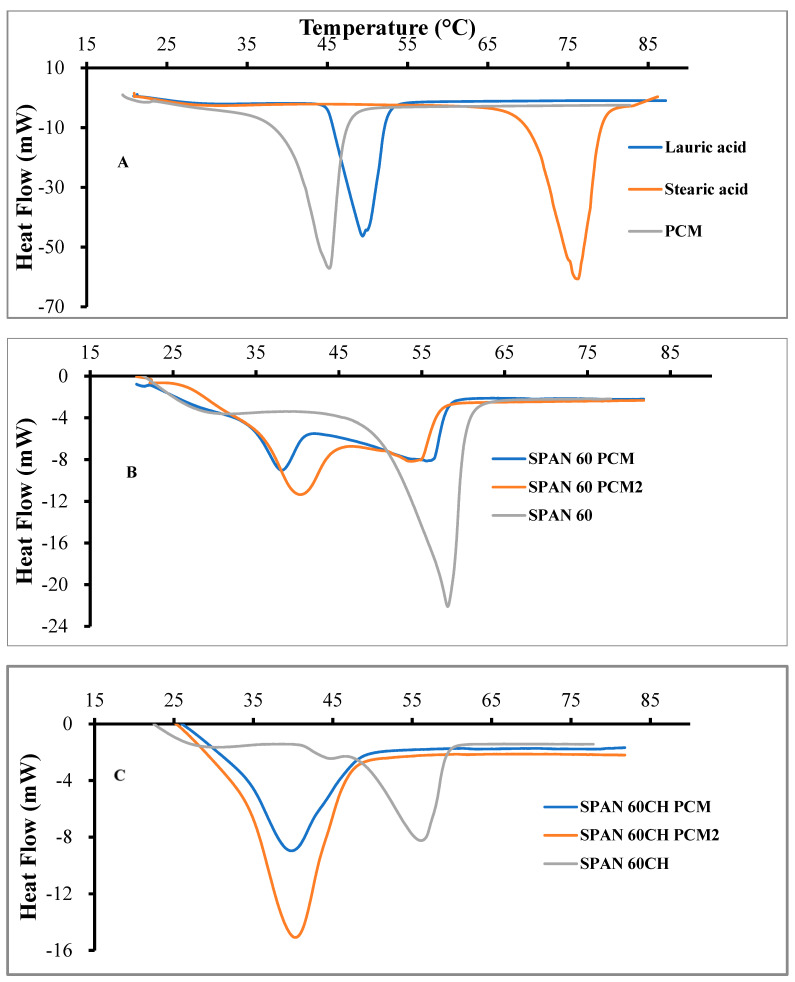
DSC thermograms of lauric acid, stearic acid and their PCM physical mixture (**A**), Span 60 and Span 60PCM mixtures (**B**) and Span 60Ch and Span 60Ch PCM mixtures (**C**)**.** All scans were performed at a rate of 3 °C/min over a temperature range of 20–85 °C under a purge of nitrogen.

Next, we used differential scanning calorimetry (DSC) to evaluate the response of the developed formulation to temperature, and the melting profiles for all the samples are shown in Figure 3.

Figure 3A shows the DSC profile corresponding to the pure fatty acid and its mixture. As expected, the melting point of the PCM is lower than that corresponding to the two components. The melting point of the SPAN60 and SPAN60CH was around 55 °C, and the introduction of the PCM in the vesicle bilayer of these niosomes conferred in them a melting point of around 40 °C (Figure 3B,C).

### 3.2. In Vitro Release Studies

The thermoresponsive properties of the developed nanodevices were investigated by performing drug release experiments at two different temperatures, corresponding to physiological (37 °C) and hyperthermic temperature (42 °C). Ideal drug delivery systems for antibiotic delivery should ensure no premature drug leakage during blood circulation, but quick release at the target sites.

Preliminary studies were conducted to achieve the best surfactant/PCM ratio that produces a formulation with suitable thermoresponsive properties. The inclusion of PCMs in vesicles prepared without cholesterol conferred to the systems a thermoresponsiveness dependent on the amount of eutectic mixture added in the bilayer. As shown in Figure S1, SP60PCM2TC, with the higher amount of PCM, achieved a total release of the entrapped drug after only 4 h at hyperthermic temperatures, while SP60PCM released 93% of the TC after 7 h. The release observed at 37 °C for these formulations was slower, at about 60% at 24 h for both systems. These results confirm our hypothesis that the inclusion of a eutectic mixture in a vesicle bilayer with a melting point of 39 °C allows us to achieve a control of release dependent on temperature. Additionally, a significant leakage of TC at 37 °C was already observed, making these devices less efficient and increasing the risk of systemic toxicity. Therefore, we decided to incorporate cholesterol in the bilayer, because of its ability to increase niosomes’ stability and decrease the leakage of encapsulated content in the bloodstream [33,34].

**Figure 4:**
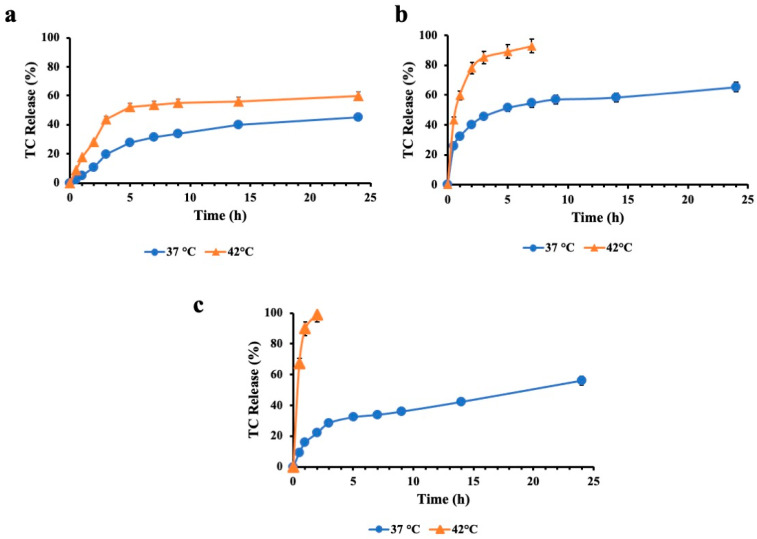
In vitro cumulative release profile of TC from SP60CH (**a**), SP60CHPCM (**b**) and SP60CHPCM2 (**c**) at physiological (37 °C) and hyperthermic temperatures (42 °C). Results are reported as mean values of three measurements.

Figure 4 shows the TC release experiments corresponding to the niosomes containing cholesterol. We investigated if the cholesterol incorporation affects the thermoresponsive properties of the developed niosomes. As shown in Figure 4, the TC release profile from SP60CH vesicles shows only a little increase with the temperature change from 37 to 42 °C. The cumulative release of TC is, respectively, 45.21 and 59.72% at 37 °C and 42 °C after 24 h. On the contrary, a significant increase in release was observed for vesicles that contain a PCM mixture in their bilayer. SP60CHPCM and SP60CHPCM2 achieved a release equal to 65.40 and 54.04% in 24 h at physiological temperatures. When the temperature increased to 42 °C, a fast and rapid release occurred, resulting in the release of the total amount of drug encapsulated in only 7 and 2 h, respectively, for SP60CHPCM and SP60CHPCM2. Consequently, a clear temperature-dependent release pattern was observed, highlighting the nanocarrier’s stability at physiological temperatures and the increased release ability at phase-transition temperatures, enhancing the local drug concentration at the infection site. So, cholesterol incorporation in the bilayer did not influence the temperature-triggered release properties of the developed vesicle, but limited the TC release under physiological conditions. The best release profile was observed for SP60PCM2 that was more stable at 37 °C and released the drug rapidly when the temperature was higher than the phase-transition temperature. Because of the obtained results, stability studies and antimicrobial activity were conducted only considering SP60PCM2 vesicles.

The results of TC release at the two different temperatures were then fitted into several kinetics models and linear forms for understanding the mechanism of drug release. We summarized all the kinetic parameters obtained in Table S1, and the results demonstrated that only the Peppas–Sahlin and Weibull models show acceptable fitting results. Moreover, it was found that TC release from non-thermoresponsive systems (SP60 and SP60CH) was best explained by the Peppas–Sahlin model. The values of Adj. R-Square, equal to 0.99647, and the residual sum of squares, equal to 0.00176, were, respectively, higher and smaller than those of the other models, as described in Table S1. Conversely, the pattern of release from vesicles that present in their bilayer the thermoresponsive PCM mixture at 42 °C, in addition to the Peppas–Sahlin model, were also best fitted by the Weibull model, as suggest by the high value of Adj. R-Square and the very low residual sum of squares data.

### 3.3. Stability Studies

The stability of niosomes is important for assessing their potential applications. In fact, one of the many problems with liposomes is that they are not stable aggregates, given the low chemical stability of phospholipids [35]. Stability studies of the developed vesicles stored at room temperature were carried out for 3 months and highlighted that the vesicles were stable at room temperature, and no sedimentation, creaming or flocculation was observed. To gain more detailed information, the changes in particle size, PDI values and zeta potential were monitored. As reported in Table 4, only small variations in particle size and EE% throughout 3 months of storage occurred, suggesting that these formulations are suitable for drug delivery applications

**Figure 5:**
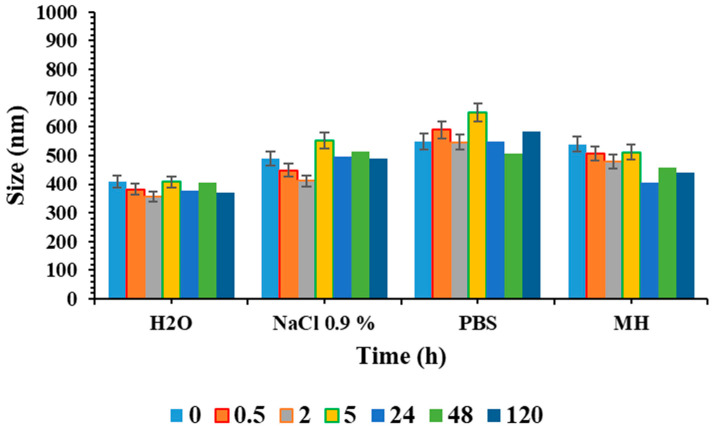
Colloidal stability of SPAN60CHPCM at 37 °C and at different times (0, 0.5, 5, 24, 48 and 120 h) of incubation in H_2_O, PBS, NaCl 0.9% and MH, Mueller–Hinton broth. Replicates of three different batches were considered, and the results are mean values ± SD.

The stability studies of niosome formulations were also assessed in various medium simulant biological conditions (Figure 5, Figures S2 and S3), since complex mixtures with a certain pH, ionic strength and often the presence of organic matter can influence the stability and, thereby, alter the efficacy.

No significant alterations in dimensions and ζ-potential within 120 h were observed in the different conditions tested. However, niosome diameter in PBS and NaCl 0.9% was slightly larger than that in water, which can be ascribed to the salt absorption on the particle surface. So, it is possible to conclude that niosomes are stable in physiological conditions and the culture medium used for antibacterial assays. This suggests that the system is stable, and is therefore suitable for antibacterial applications.

### 3.4. Antimicrobial Activity

The antimicrobial activity (MIC and MBC) of empty and TC-loaded niosomes were evaluated at two different temperatures (37 °C and 42 °C) against some representative Gram-positive and Gram-negative bacteria. This study was carried out to investigate the antimicrobial performance of the developed nanodevices and to identify if there would be some enhancement of antibacterial efficacy with temperature.

The experiments were carried out only on SP60CHPCM2, which, as reported above, presents better thermoresponsive properties, and the results are shown in Table 5 and Table 6. For comparison purposes, the MIC and MBC values of free TC and empty niosomes were also measured.

At the higher concentration tested, 2970 μM, the empty niosomes did not show antimicrobial activity against any of the tested bacteria. Only a weak activity was observed at 42 °C against some of the Gram-positive microorganisms at very high concentrations, possibly due to the lauric acid presence in the vesicle bilayer, which has been reported to have antimicrobial properties [36]. Notice that, at 42 °C, the fatty acid state has changed; this can give rise to a better bioavailability of this compound and consequently to a better antimicrobial activity. The experimental results in our study confirmed the antimicrobial activity of free TC against Gram-positive and Gram-negative bacteria with MIC values in the range of 0.6–30 μM, in accordance with that reported in the literature [37,38]. A very big MIC value against *S. epidermidis* was recorded, which agrees with previous studies that observed that, compared with other antibiotics, tetracyclines exhibited low antimicrobial effectivity against different *S. epidermidis* strains [39]. In fact, Aelenei et al. [40] reported that *S. epidermidis ATCC* 12228, the strain used in this work, is resistant to this antibiotic. The MBC values of TC are much higher than the MICs. The mode of action of tetracycline implies a binding to the bacterial ribosome, and thereby interference with protein translation. The result of this type of interaction is generally bacteriostatic rather than bactericidal [41]. At 37 °C, it was observed that TC encapsulated in niosomes maintained its antimicrobial efficiency against the tested bacteria. Even a slightly better inhibition effect was observed against BS and SE strains, with an MIC reduction from 1.62 to 0.77 and from 207.94 to 112.81, respectively (Table 5). Instead, a significant decrease in MBC values was observed for TC-loaded niosomes, unlike the ones obtained with free drugs against all of the tested bacteria, except for LM, indicating a good ability to completely kill bacteria (Table 6). For example, the MBC value of TC-loaded niosomes at 37 °C against *S. aureus* (7.05) was considerably lower than that of the free drug, equal to 103.97 μM. This enhanced antibacterial activity may be due to a change in the mode of action of the encapsulated TC against bacteria due to the better permeability and fusogenic properties of niosomes and/or the interaction of the other niosome components. Indeed, it has been demonstrated that the encapsulation of antibiotics in vesicles can improve their pharmacokinetic profiles and increase their accumulation at the infection site, minimizing their cytotoxicity and protecting them from peripheral degradation [42].

Moreover, the developed niosomes showed an interesting temperature-dependent antibacterial activity against some bacterial strains. In fact, the temperature increase to 42 °C further improves the activity of the niosomes against *S. Epidermis*, with a lower MIC value equal to 14.10 μM, unlike that obtained at 37 °C, which was equal to 112.81 μM. A moderate temperature-dependent trend in antibacterial activity was also observed for EC, EF and AB strains.

For example, MIC values for *E. faecalis*, *E. coli* and *A. baumannii* at 37 °C were 28.20, 7.05 and 7.05, and were reduced to 14.01, 3.52 and 3.52, respectively, by temperature increase. It is noticeable that in using this niosomial formulation, one resistant bacteria has become a sensitive one. This shows that through this type of technology, it is possible to reuse antibiotics that were no longer effective against certain resistant bacteria; so, this could be an interesting approach to combat the emergence of resistant bacteria. This shows again that the encapsulation of TC in thermoresponsive niosomes also permitted the antimicrobial activity of the drug to be exerted at a lower concentration than that of the free drug, without affecting the treatment outcomes and reducing the side effects.

The experimental results demonstrate that the inclusion of a eutectic mixture in the vesicle bilayer not only affects drug release but also affects antibacterial activity. The enhanced activity at 42 °C of the TC-loaded niosomes for some of the tested bacteria can be ascribed to a better ability to release their content at temperatures above the melting point of the eutectic mixture, increasing drug concentrations at the infection site and offering a better killing effect. Taken together, encapsulating the antibiotic into a thermoresponsive nanocarrier provides good biosafety, prevents premature release, allows for a more precise release of antibiotics and eliminates bacteria more effectively, which makes these devices appealing for further development into a clinical agent to prevent serious bacterial infections.

In this regard, the literature contains a lot of formulations containing antibiotics encapsulated in liposomial formulations that improve the antimicrobial activity of the drug [43]. In this respect, a TC-liposomial formulation has been already approved by the FDA to treat the *Chlamydia trachomatis* [44]. However, liposomes show certain disadvantages: low solubility, a short half-life, low chemical phospholipid stability and high production cost. Gentamicin-loaded niosomes were also prepared using a non-ionic surfactant (Tween 60, tween 80 or Brij 35), cholesterol and a negative charge inducer, dicetyl phosphate. Niosomes composed of Tween 60, cholesterol and dicetyl phosphate were the most effective in terms of prolonged in vitro drug release [45].

In this work, we have prepared, for the first time, a stable thermosensitive tetracycline-niosomial system using economical starting materials. It has been assessed that the release of the antibiotic at 42 °C is higher than that at 37 °C, which could improve the effectivity of antibiotics against some resistant bacteria.

To evaluate the biosafety of the newly developed nanocarriers (empty and TC-loaded), a hemolysis assay was performed on human erythrocytes. It was found that both empty and TC-loaded vesicles were hemocompatible, causing a negligible release of hemoglobin from erythrocytes, even at the highest concentration tested. We observed only a slight hemolytic activity, equal to 11.37 ± 1.99% and 19.97 ± 1.99% of empty and drug-loaded systems, at the concentration of 119 μg/mL of LA. These values indicate that the therapeutic index of these systems (hemolysis/MIC ratio) is very high, which indicates the high biocompatibility of these formulations. The non-toxicity of free and TC-loaded niosomes makes these new antimicrobial systems highly desirable as safe nanocarriers for biomedical applications.

## 4. Conclusions

In the present study, we developed a novel platform based on thermoresponsive niosomes with the purpose of controlling TC delivery in the treatment of bacterial infections. The systems produced showed good physicochemical properties and entrapment efficiency, stability and tunable thermoresponsive properties. The developed niosomes were able to prevent the premature release of TC in physiological conditions, while they have shown a fast release at 42 °C. This thermoresponsive release was also associated with an enhanced antibacterial efficacy in hyperthermic conditions that allows us to use low antibiotic doses, maintaining therapeutic outcomes and reducing the adverse side effects of common antimicrobial agents. The present smart platform may hold promise for the fight against various bacteria-induced diseases.

## Figures and Tables

**Table 1:** Kinetic model used to analyze the data of TC release from the developed thermosensitive niosomes.

Release Kinetic Model	Mathematical Equation
First order	Q/Q_0_ = 1 − e^kt^
Korsmeyer–Peppas	Q/Q_0_ = k t^n^
Peppas–Sahlin	Q/Q_0_ = K_1_t^m^ + k_2_ t^2m^
Weibull	Q/Q_0_ = 1 − e ^(−b × t^a^)^

**Table 2:** Composition (molar ratio) and physicochemical characterization of niosomes in terms of hydrodynamic diameter, P.I. and Z-potential. All formulations were prepared at 1 × 10^−2^ M of total lipid concentration. Values represent means ± S.D. (*n* = 3).

Formulation	Span60	Chol	PCM	Size (nm)	P.I.	Z-Potential (mV)
SP60	1	-	-	348.3 ± 6.8	0.250	−28.3 ± 0.87
SP60PCM	1	-	0.5	327.3 ± 3.6	0.220	−30.6 ± 0.92
SP60PCM2	1	-	0.7	270.0 ± 4.5	0.237	−34.8 ± 0.83
SP60CH	0.5	0.5	-	311.1 ± 4.9	0.270	−35.7 ± 0.76
SP60CHPCM	0.5	0.5	0.5	270.7 ± 8.7	0.281	−37.7 ± 1.18
SP60CHPCM2	0.5	0.5	0.7	244.9 ± 6.8	0.225	−29.3 ± 0.84

**Table 3:** Physicochemical characterization of niosomes loaded with TC in terms of hydrodynamic diameter, P.I., Z-potential and encapsulation efficiency (EE%). Values represent means ± S.D. (*n* = 3).

Formulation	Size (nm)	P.I.	Z-Potential (mV)	EE%
SP60TC	375.0 ± 7.9	0.175	−26.1 ± 0.58	66.36 ± 3.79
SP60PCMTC	352.5 ± 5.7	0.192	−23.5 ± 1.97	53.44 ± 6.57
SP60PCM2TC	355.3 ± 8.5	0.245	−23.0 ± 0.889	81.29 ± 8.95
SP60CHTC	488.3 ± 4.3	0.184	−26.1 ± 0.520	71.84 ± 5.36
SP60CHPCMTC	316.4 ± 7.5	0.263	−27.9 ± 0.503	54.86 ± 6.82
SP60CHPCM2TC	353.3 ± 9.5	0.275	−26.6 ± 0.611	66.78 ± 4.77

**Table 4:** Storage stability of SPAN60CHPCM2 stored at room temperature for up to 3 months, evaluated measuring diameter, P.I., ξ-potential and D.L. at specific time points. Data are expressed as means of three different experiments ± SD.

Time (Days)	Size (nm)	P.I.	Z-Potential (mV)	EE%
0	353.3 ± 9.5	0.275	−26.6 ± 0.61	66.78 ± 4.77
15	326.4 ± 9.8	0.255	−27. 1 ± 1.01	65.34 ± 3.44
30	391.0 ± 6.3	0.276	−26.0 ± 0.52	65.11 ± 3.75
45	382.8 ± 8.7	0.310	−27.2 ± 0.52	64.99 ± 6.78
60	379.4 ± 3.6	0.286	−25.5 ± 0.42	62.59 ± 5.85
90	384.9 ± 4.4	0.274	−24.5 ± 0.53	63.01 ± 4.98

**Table 5:** MIC values (μM) of free TC, empty and TC-loaded niosomes: BS (*B. subtilis*), SE (*S. epidermidis*), SA (*S. aureus*), LM (*L. monocytogenes*), EF (*Enterococcus faecalis*), EC (*E. coli*), AB (*A. baumannii*) and KA (*Klebsiella pneumoniae*) at 37 °C and 42 °C.

MIC	37 °C			42 °C		
	SP60CHPCM2	SP60CHPCM2 TC	TC	SP60CHPCM2	SP60CHPCM2 TC	TC
BS	>2970	0.77	1.62	371.26	0.77	1.62
SE	>2970	112.81	207.94	371.26	14.10	103.97
SA	>2970	3.52	3.25	742.57	3.52	3.25
LM	>2970	7.05	3.25	>2970	3.108	1.62
EF	>2970	28.20	25.99	>2970	14.01	12.99
EC	>2970	7.05	6.49	>2970	3.52	6.49
AB	>2970	7.05	6.49	>2970	3.52	3.25
KA	>2970	14.1	6.49	>2970	14.1	6.49

**Table 6:** MBC values (μM) of free TC, empty and TC-loaded niosomes: BS (*B. subtilis*), SE (*S. epidermidis*), SA (*S. aureus*), LM (*L. monocytogenes*), EF (*Enterococcus faecalis*), EC (*E. coli*), AB (*A. baumannii*) and KA (*Klebsiella pneumoniae*) at 37 °C and 42 °C.

MBC	37 °C			42 °C		
	SP60CHPCM2	SP60CHPCM2 TC	TC	SP60CHPCM2	SP60CHPCM2 TC	TC
BS	>2970	0.77	1.62	742.57	0.77	>6.49
SE	>2970	112.81	>415.88	1485.14	>49.74	>103.97
SA	>2970	7.05	>103.97	1485.14	>24.87	103.97
LM	>2970	7.05	6.49	>2970	3.108	3.25
EF	>2970	28.20	>207.94	>2970	>49.74	51.98
EC	>2970	7.05	>25.99	>2970	>24.87	>25.99
AB	>2970	7.05	>25.99	>2970	24.87	12.99
KA	>2970	14.1	>51.98	>2970	>49.74	>25.99

## Data Availability

The data presented in this study are available within this article.

## Supplementary Materials

The following supporting information can be downloaded at: https://www.mdpi.com/article/10.3390/pharmaceutics16070909/s1, Figure S1. In vitro cumulative release profile of TC from SP60 (A), SP60PCM (B) and SP60PCM2 (C) at physiological (37 °C) and hyperthermic temperature (42 °C). Figure S2. Measurements of polydispersity index (PDI) of SPAN60CHPCM at 37 °C and at different time (0, 0.5, 5, 24, 48, 120 h) of incubation in H_2_O, PBS, NaCl 0.9% and MH. Replicates of three different batches were considered and results are mean values ± SD. Figure S3. Measurements of Z-potential (mV) of SPAN60CHPCM at 37 °C and at different time (0, 0.5, 5, 24, 48, 120 h) of incubation in H_2_O, PBS, NaCl 0.9% and MH. Replicates of three different batches were considered and results are mean values ± SD. Table S1. The release kinetics model fitting parameters of TC release from the developed formulations.
