# Supplementary material for: Design of Thermosensitive Niosomes by Eutectic Mixture of Natural Fatty Acids
Source: Pharmaceutics. 2024 Jul 7;16(7):909. doi: 10.3390/pharmaceutics16070909 (PMC11279573; doi:10.3390/pharmaceutics16070909)
Supplement: Supplementary file 1 [file pharmaceutics-16-00909-s001.zip › pharmaceutics-3082676-supplementary.pdf]

DESIGN OF THERMOSENSITIVE NIOSOMES BY EUTECTIC MIXTURE OF NATURAL FATTY ACIDS.

Elisabetta Mazzotta[1*], Martina Romeo[1], Zakaria Hafidi[2], Lourdes Perez[2], Ida Daniela Perrotta[3], Rita Muzzalupo[1*]

1 Department of Pharmacy, Health and Nutritional Sciences, University of Calabria, via P. Bucci, 87036 Arcavacata di Rende (CS), Italy;

2 Department of Surfactants and Nanobiotechnology, Institute for Advanced Chemistry of Catalonia (IQAC-CSIC), 08034 Barcelona, Spain

3 Centre for Microscopy and Microanalysis (CM2), Department of Biology Ecology and Earth Sciences, University of Calabria, 87036 Arcavacata di Rende, Italy

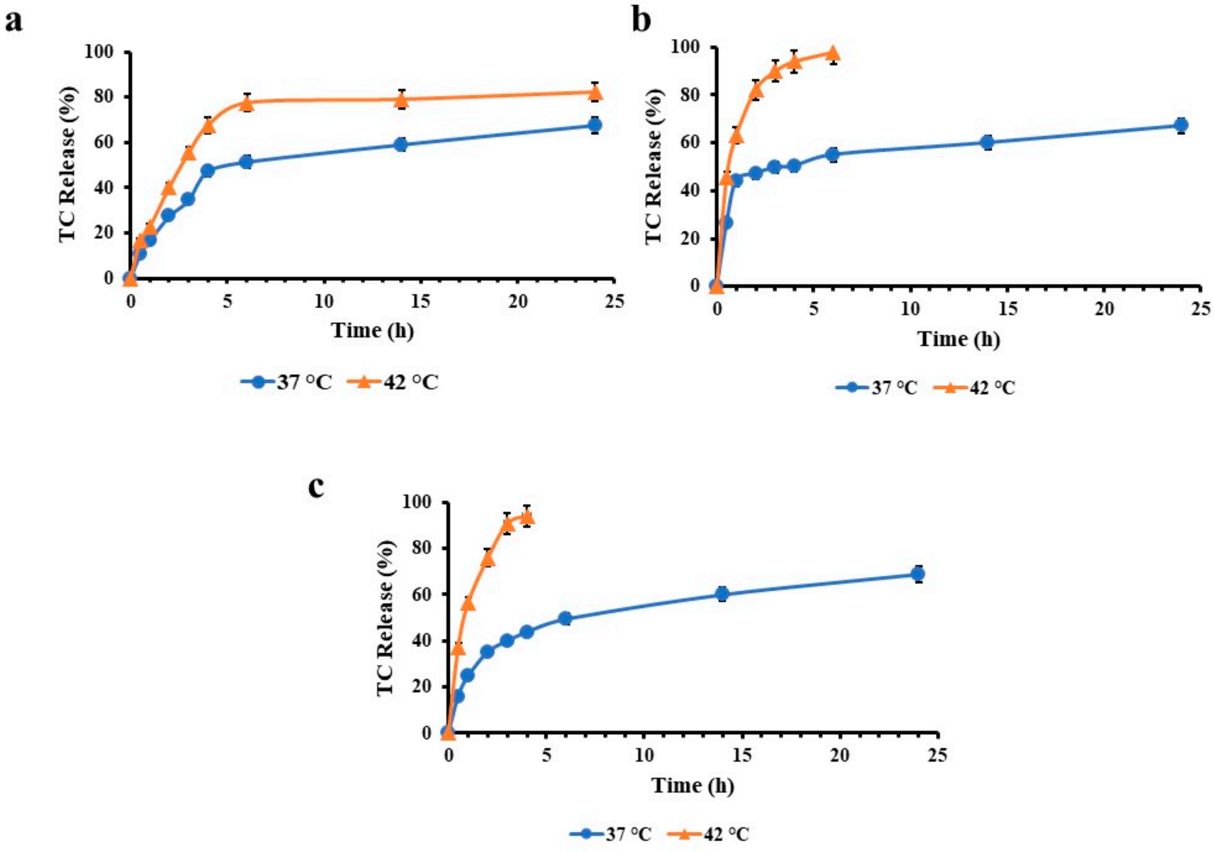

Figure S1. *In vitro* cumulative release profile of TC from SP60 (A), SP60PCM (B) and SP60PCM2 (C) at physiological (37°C) and hyperthermic temperature (42°C).

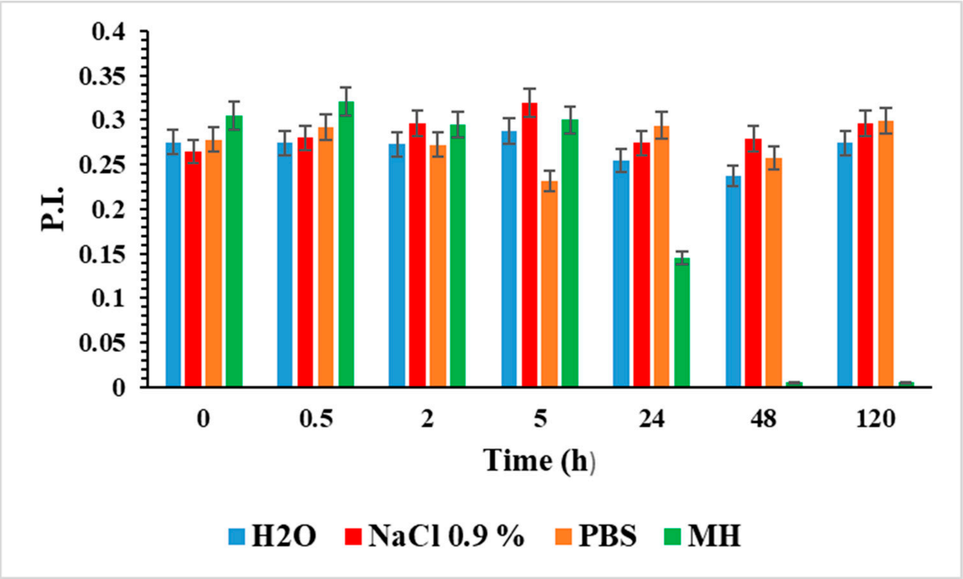

Figure S2. Measurements of polydispersity index (PDI) of SPAN60CHPCM at 37°C and at different time (0, 0.5, 5, 24,48,120 h) of incubation in H_2O, PBS, NaCl 0.9% and MH. Replicates of three different batches were considered and results are mean values ± SD.

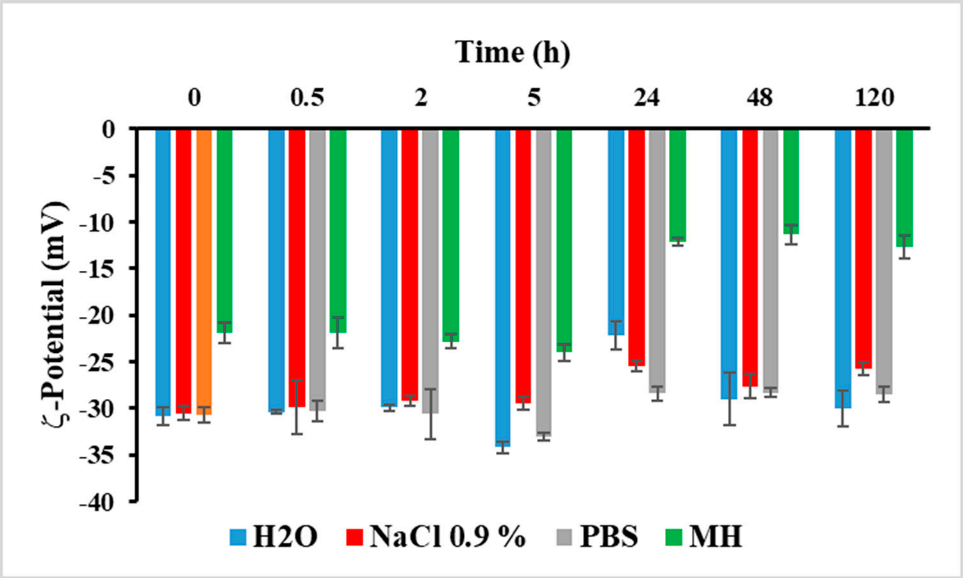

Figure S3. Measurements of Z-potential (mV) of SPAN60CHPCM at 37°C and at different time (0, 0.5, 5, 24,48,120 h) of incubation in H_2O, PBS, NaCl 0.9% and MH. Replicates of three different batches were considered and results are mean values ± SD.

Table S1. The release kinetics model fitting parameters of TC release from the developed formulations.

SP60					
**KINETIC MODEL**	**T**	**R^2**	**ADJ. R-SQUARE**	**PEARSON'S R**	**RESIDUAL SUM OF SQUARE**
**First order**	37°C	0.80081	0.76097	-0.89488	0.14926
	42°C	0.61806	0.54167	-0.78617	0.82589
**Korsmeyer-Peppas**	37°C	0.91327	0.89593	0.95565	0.22795
	42°C	0.84664	0.81597	0.92013	0.36034
**Peppas-Sahlin**	37°C	0.98784	0.98297	-	0.01431
	42°C	0.99748	0.99647	-	0.00176
**Weibull**	37°C	0.94415	0.93299	0.97168	0.22089

	42°	0.88818	0.86582	0.94243	0.50738
**SP60PCM**					
**First order**	37°C	0.75889	0.71066	-0.87114	0.08458
	42°C	0.98567	0.9809	-0.99281	0.05592
**Korsmeyer-Peppas**	37°C	0.82995	0.79594	0.91102	0.08396
	42°C	0.94688	0.9336	0.97308	0.02355
**Peppas-Sahlin**	37°C	0.98771	0.9814	-	0.02313
	42°C	0.99969	0.99954	-	0.00121
**Weibull**	37°C	0.87774	0.85329	0.93688	0.11167
	42°C	0.99975	0.99968	0.99975	0.00060
**SP60PCM2**					
**First order**	37°C	0.8852	0.86224	-0.94085	0.07219
	42°C	0.98567	0.9809	-0.99281	0.05592
**Korsmeyer-Peppas**	37°C	0.9461	0.93532	0.97268	0.07471
	42°C	0. 984	0.97338	0.98997	0.01201
**Peppas-Sahlin**	37°C	0.99921	0.99889	-	0.00101
	42°C	0.99858	0.99764	-	0.00387
**Weibull**	37°C	0.97771	0.97325	0.98879	0.05084
	42°C	0.99368	0.99158	0.99684	0.01401
**SP60CH**					
**First order**	37°C	0.78604	0.74325	-0.88659	0.05482
	42°C	0.51704	0.42045	-0.71906	0.28478
**Korsmeyer-Peppas**	37°C	0.88692	0.86431	0.94177	0.79899
	42°C	0.80612	0.76735	0.89784	0.59907
**Peppas-Sahlin**	37°C	0.96181	0.94653	-	0.01653
	42°C	0.97669	0.96736	-	0.02855
**Weibull**	37°C	0.90512	0.886887	0.9517	0.78060
	42°C	0.833	0.7996	0.91269	0.73374
**SP60CHPCM**					
**First order**	37°C	0.75812	0.70974	-0.8797	0.09704
	42°C	0.94298	0.92873	-0.97107	0.17698
**Korsmeyer-Peppas**	37°C	0.94688	0.93626	0.97308	0.03264
	42°C	0.92584	0.91842	0.96221	0.03216
**Peppas-Sahlin**	37°C	0.99782	0.99695	-	0.00333
	42°C	0.99029	0.99148	-	0.04439
**Weibull**	37°C	0.96268	0.95521	0.98116	0.04082
	42°C	0.99687	0.99583	0.99843	0.00134
**SP60CHPCM2**					
**First order**	37°C	0.91163	0.89396	-0.9548	0.02951
	42°C	0.92921	0.91152	-0.96396	0.20456
**Korsmeyer-Peppas**	37°C	0.95525	0.9463	0.97737	0.09069
	42°C	0.98445	0.98056	0.99219	0.01046
**Peppas-Sahlin**	37°C	0.99748	0.99647	-	0.00176
	42°C	0.99986	0.99972	-	0.00016
**Weibull**	37°C	0.97506	0.97007	0.98745	0.06977
	42°C	0.9966	0.99547	0.9983	0.00146
